# Prevalence of mastitis pathogens in South African pasture-based and total mixed ration-based dairies during 2008 and 2013

**DOI:** 10.4102/ojvr.v85i1.1482

**Published:** 2018-05-31

**Authors:** David Blignaut, Peter Thompson, Inge-Marié Petzer

**Affiliations:** 1 Department of Production Animal Studies, University of Pretoria, South Africa

## Abstract

Recent years have seen a change in the relative prevalence of environmental and contagious intramammary pathogens, as well as a change in the relative number of total mixed ration (TMR)-based and pasture (PAS)-based dairies in South Africa. The objectives of the study were to determine and compare the prevalence of mastitis pathogens in TMR and PAS dairies in South Africa during 2008 and 2013; furthermore, the within-herd prevalence of *Streptococcus uberis* in *Str. uberis*-positive herds was determined and compared. The prevalence of each pathogen, as well as the within-herd prevalence of *Str. uberis*, were compared between the two years and the two management systems using bacterial culture results from routinely collected composite cow milk samples submitted to the Onderstepoort Milk Laboratory, Faculty of Veterinary Science, University of Pretoria. Coagulase-negative staphylococci had the highest prevalence in both TMR and PAS dairies for both 2008 (29.60% [95.00% CI: 28.80% – 30.40%] and 26.90% [95.00% CI: 25.50% – 28.30%], respectively) and 2013 (20.20% [95.00% CI: 19.30% – 21.10%] and 22.70% [95.00% CI: 22.20% – 23.10%], respectively), which decreased significantly from 2008 to 2013 in both TMR and PAS dairies (*p* < 0.001). *Streptococcus uberis* showed an increase in prevalence in both TMR (*p* = 0.002) and PAS dairies (*p* = 0.001) from 2008 (2.36% [95.00% CI: 2.10% – 2.65%] and 2.63% [95.00% CI: 2.16% – 3.16%], respectively) to 2013 (3.10% [95.00% CI: 2.72% – 3.51%] and 3.64% [95.00% CI: 3.45% – 3.83%], respectively). *Staphylococcus aureus* showed a significant decrease in both TMR (*p* = 0.011) and PAS (*p* < 0.001) dairies from 2008 (4.71% [95.00% CI: 4.34% – 5.10%] and 5.62% [95.00% CI: 4.94% – 6.36%], respectively) to 2013 (3.95% [95.00% CI: 3.52% – 4.40%] and 1.71% [95.00% CI: 1.58% – 1.84%], respectively). The median within-herd prevalence of *Str. uberis* for the combined dairy systems showed a significant increase from 2008 (1.72% [IQR: 0.88% – 5.00%]) to 2013 (3.10% [IQR: 1.72% – 4.70%]) (*p* < 0.001). Statistically significant differences were found in the prevalence of most of the major contagious and environmental mastitis pathogens between 2008 and 2013 and between TMR and PAS dairies. The within-herd prevalence of *Str. uberis* increased from 2008 to 2013, with the highest within-herd prevalence in PAS dairies in 2013.

## Introduction

In recent years, there has been an increase in the prevalence of environmental pathogens as a cause of intramammary infections (IMI) in dairy cattle (Bradley 2002; Charman et al. 2012; Milne et al. 2002; Oliveira et al. 2015; Petzer et al. 2009). Increases in prevalence could potentially be ascribed to improved control methods for contagious pathogens, difficulties in controlling pathogens from an environmental reservoir and the ability of *Streptococcus uberis* and *Escherichia coli* to persist in the udder (Leigh 1999; Matthews, Almeida & Oliver 1994).

*Streptococcus uberis*, being one of the important cultured pathogens in south-eastern Australia, the United Kingdom and New Zealand (Bradley 2002; Charman et al. 2012; McDougall 1998; Milne et al. 2002), showed an increase in prevalence over an 11-year study period (1996–2007) in South African dairies (Petzer et al. 2009). A further increase in prevalence of environmental IMI is evident in data collected from cow milk samples cultured between 2008 and 2012 (I.M. Petzer [University of Pretoria] unpubl. data, 2012). During the same period, there was an overall increase in the number of pasture (PAS)-based dairies compared to dairies feeding total mixed rations (TMRs) in South Africa (Lactodata 2014). In 2015, approximately 80% of all milk was produced in the coastal regions of South Africa, with the majority of those dairies making use of pastures (Lactodata 2015). Furthermore, the spreading of dairy effluent (manure) to fertilise pastures has become more popular in many of these dairies. This management practice has not been investigated as a potential risk factor for the prevalence of *Str. uberis* in South Africa.

Shum et al. (2009) reported that the pathogen prevalence profile from earlier Australian studies that focussed on predominantly PAS-based dairies differed from their study that focussed on high-producing, intensively managed dairy farms in New South Wales, Australia. Environmental pathogens, in particular *Str. uberis,* had the highest prevalence in the PAS-based dairies, compared to *E. coli* that was more frequently isolated in the high-producing, intensively managed dairy farms.

The prevalence of mastitis pathogens is changing in South Africa, as shown by Petzer et al. (2009). It was reported by Motaung et al. (2017) that in South Africa, fewer than six different pathogens are commonly reported as mastitis - causing pathogens. It was also interesting to note that there was no reported data on pathogen prevalence in many other African countries (Motaung et al. 2017). There is thus a need to report data from an African context.

The objective of this study was to use retrospective culture results from composite cow milk samples to estimate and compare the prevalence of mastitis pathogens in different management systems (TMR and PAS) between 2008 and 2013. Furthermore, the within-herd prevalence for *Str. uberis* was estimated and compared in the different systems between 2008 and 2013, as it was deemed important to determine whether or not the number of herds that were regarded as *Str. uberis* - positive were on the rise.

## Materials and methods

This was a retrospective cross-sectional study using available bacterial culture results of composite cow milk samples from dairy farms submitted during 2008 and 2013. Culture results from 2008 were used to follow up on a previous study by Petzer et al. (2009), in which culture results up until 2007 were used. At the time of the study, the 2013 culture results were the most recent full set of available results, and it was decided to conclude the study with these results from 2013. The samples were submitted to the Onderstepoort Milk Laboratory (OML), Faculty of Veterinary Science at the University of Pretoria. The OML has over 950 registered clients, including referring veterinarians. A total of 46 067 and 130 870 cow milk samples (including quarter and composite cow milk samples) were submitted during 2008 and 2013, respectively. The OML received milk samples from 94 and 121 dairy producers during 2008 and 2013, respectively. In June 2017, there were 1503 registered dairy producers in South Africa (Lactodata 2017).

### Study population

The study population was defined as all lactating dairy cows from dairies that submitted composite cow milk samples to OML during 2008 and 2013 for microbial culture and identification, as well as somatic cell count (SCC) determination. The microbial cultures were done for routine udder health monitoring within the herds, regardless of the clinical mastitis status of the herd. Differentiation was made between TMR and PAS dairies. Total mixed ration dairies were defined as dairies where the main feeding system was based on a complete mixed ration. This system could be either free stall barns or open outside paddocks; there was no differentiation made between the two. Pasture-based dairies were defined as dairies where the main feeding system was pasture-based. Within this system, lactating cows might have received additional feed, such as silage, during the dry season.

The samples were received from dairies situated across South Africa, with more than 50% from KwaZulu-Natal, and the Eastern Cape and Western Cape provinces. Approximately 81% of the total milk in South Africa is produced in these three provinces (Lactodata 2015).

The composite cow milk samples were collected aseptically by trained animal technicians, referring veterinarians and farmers from foremilk of the four quarters of the same cow. Each sample was identified with the corresponding cow number. Milk samples were shipped on ice to reach the laboratory within 48 h.

### Laboratory culture of composite cow milk samples

Milk was plated out on bovine blood tryptose agar (BTA) (Oxoid, supplied by Quantum Biotechnologies [Pty] Ltd, Ferndale, South Africa). Inoculated agar plates were incubated at 37 °C ± 1 °C and plates were examined after 18–24 h and 48 h. In addition, all samples from clinical mastitis cases were enriched, using 0.5 mL of milk added to 5.0 mL Brain heart media and incubated at 37 °C for 18–24 h. Isolated bacteria were identified in accordance with standard laboratory milk culture methodology based on colony morphology, as described by the IDF Document 132 of 1981. Tests used included Staphylase, Strepkit, Catalase, DNase, KOH (Oxoid, supplied by Quantum Biotechnologies [Pty] Ltd, Ferndale, South Africa), Maltose (Merck NT Laboratory Supplies, Halfway House, South Africa) and API^®^ 20E and Staph API^®^ (bioMérieux South Africa [Pty] Ltd, Randburg, South Africa).

*Staphylococcus aureus* isolates were further identified by phage typing (Blair & Williams 1961). Typing was performed using an international set of 23 phages. The strains were typed as one of four groups or as non-allocated. All *S. aureus* isolates that were identified as being from phage group 3 were indicated by the abbreviation STH, whereas all those that were not from this group were identified as STA.

A diagnosis was only made when two or more colonies were present on a plate. In this study, the presence of a bacterial species (more than two colonies) was regarded as an intramammary infection. Milk samples were identified as being contaminated (CU) when three or more different colony types were present on a plate.

### Data analysis

Data from 2008 and 2013 were transferred in a comma–separated value (CSV) file from the OML MSD^®^ program to a Microsoft Excel spreadsheet. Data consisted of the following variables: producer code, date cultured, bacteria cultured, management system (TMR or PAS) and cow number. Records that had missing values for any of these variables or that had both management systems were excluded from the study.

Retrospective data from complete herds for udder health investigations and routine culture were used in the study. Partial herd test results were excluded from the study to reduce the bias towards the testing of cows showing clinical mastitis or towards specific bacteria cultured during the follow-up of a mastitis outbreak in a herd. If a herd was tested more than once during a year, one of the herd tests was selected randomly and the other samples were excluded from the data set. All quarter milk samples were excluded. The final data set consisted of 16 415 and 45 815 composite cow milk samples from 2008 and 2013, respectively.

The prevalence of bacteria, which included contaminant bacteria, was calculated, overall and by year and system, with exact 95% confidence intervals, and was compared between years and management systems using Fisher’s exact test.

For each of the bacteria, a multiple logistic regression model was then used to estimate the association of year (2008 versus 2013) and system (TMR versus PAS) with the odds of culturing the organism, while adjusting for confounding. Herds was included as a random effect. The interaction between year and system was also assessed.

For *Str. uberis*-positive herds, the effect of year (2008 versus 2013) and system (TMR versus PAS) on the within-herd prevalence of *Str. uberis* was estimated using multivariable Poisson regression. The outcome variable was the number of positive cultures of *Str. uberis*, and the total number of samples tested was included as an exposure variable. Herds was included as a random effect. The interaction between year and system was also assessed. Data analysis was performed using Stata 12.1 (StataCorp, College Station, TX, USA); statistical significance was assessed at *p* < 0.05.

### Ethical considerations

Ethical approval for this study was obtained from the University of Pretoria Animal Ethics Committee (No: V015-15).

## Results

### Prevalence of mastitogenic pathogens

With a final data set consisting of 62 230 (16 415 in 2008 and 45 815 in 2013, respectively) samples from 123 herds – 83 herds from PAS dairies and 40 herds from TMR dairies – comparisons of the prevalence of each pathogen between the systems and the years were summarised, as shown in Table 1.

**TABLE 1:** Prevalence of mastitis pathogens by year (2008 and 2013) and system (total mixed ration- and pasture-based) in South African dairy herds.

Pathogen	2008				2013			
	TMR (*n* = 12 269)		PAS (*n* = 4146)		TMR (*n* = 7752)		PAS (*n* = 38 063)	
	Prevalence (%)	95% CI	Prevalence (%)	95% CI	Prevalence (%)	95% CI	Prevalence (%)	95% CI
SDY	1.12	0.94–1.32	0.8	0.55–1.12	1.25	1.02–1.52†	0.64	0.56–0.72†
SUB	2.36	2.10–2.65*	2.63	2.16–3.16*	3.1	2.72–3.51*†	3.64	3.45–3.83*†
STA	4.71	4.34–5.10*†	5.62	4.94–6.36*†	3.95	3.52–4.40*†	1.71	1.58–1.84*†
STH	0.79	0.64–0.96*†	2.87	2.38–3.42*†	0.03	0.00–0.09*	0.09	0.06–0.13*
SAG	1.01	0.84–1.20*†	1.62	1.25–2.05*†	8.02	7.43–8.65*†	0.76	0.67–0.85*†
CNS	29.6	28.8–30.4*†	26.9	25.5–28.3*†	20.2	19.3–21.1*†	22.7	22.2–23.1*†
SFA	0.9	0.74–1.08*	0.75	0.51–1.06*	0.37	0.25–0.54*†	0.14	0.10–0.18*†
CU	13.7	13.1–14.4*	13.2	12.1–14.2*	16.5	15.6–17.3*†	4.9	4.69–5.12*†
OTHER	3.11	2.81–3.43*	3.59	3.05–4.21*	2.39	2.06–2.75*†	1.4	1.28–1.52*†
IMI	57.4	56.5–58.2*	57.9	56.4–59.4*	55.7	54.6–56.8*†	35.9	35.4–36.4*†

TMR, total mixed ration; PAS, pasture; SDY, *Streptococcus dysgalactiae*; SUB, *Streptococcus uberis*; STA, *Staphylococcus aureus;* STH, *Staphylococcus aureus* of suspected human origin; SAG, *Streptococcus agalactiae*; CNS, coagulase-negative staphylococci; SFA, *Enterococcus faecalis*; CU, contamination; OTHER, minor pathogens including gram-negative bacteria; IMI, intramammary infection, CI, confidence interval.

* , The same system differs between years (*p* < 0.05);

† , Systems differ within the same year (*p* < 0.05).

*Streptococcus uberis* showed a significant increase in prevalence in both TMR (*p* = 0.002) and PAS dairies (*p* = 0.001) from 2008 to 2013. In 2013, the prevalence of *Str. uberis* was higher (*p* = 0.018) in PAS dairies compared to TMR dairies. The prevalence of *S. aureus* decreased in TMR (*p* = 0.011) and PAS (*p* < 0.001) dairies from 2008 to 2013. *Staphylococcus aureus* of suspected human origin showed a decrease in prevalence in both TMR (*p* < 0.001) and PAS (*p* < 0.001) dairies from 2008 to 2013. *Streptococcus agalactiae* showed an increase (*p* < 0.001) in prevalence in TMR dairies from 2008 to 2013, with a decrease (*p* < 0.001) in PAS dairies during the same period. Coagulase-negative staphylococci (CNS) showed the highest prevalence of all the pathogens in all the four-year-system groups. There was a decrease (*p* < 0.001) in the prevalence in both TMR and PAS dairies from 2008 to 2013. Overall, the prevalence of intramammary infections decreased in both TMR (*p* = 0.023) and PAS (*p* < 0.001) dairies from 2008 to 2013 (Table 1).

*Streptococcus uberis, Str. agalactiae* and CU showed an increase in prevalence in 2013 compared to 2008 (OR > 1) (Table 2). On the contrary, *S. aureus, S. aureus* of suspected human origin, CNS, *Enterococcus faecalis*, minor pathogens including gram-negative bacteria (OTHER) and the prevalence of overall IMI were lower in 2013 (OR < 1) (Table 2).

**TABLE 2:** Comparison of mastitis pathogen prevalence between 2008 and 2013 after adjusting for system using multiple logistic regression.

Pathogen	OR†	95% CI	*p*
SDY	1.08	0.79–1.48	0.61
SUB	1.79	1.38–2.33	< 0.001
STA	0.52	0.46–0.59	< 0.001
STH	0.03	0.01–0.08	< 0.001
SAG	5.55	3.70–8.31	< 0.001
CNS	0.68	0.62–0.75	< 0.001
SFA	0.41	0.25–0.69	0.001
CU	1.24	1.09–1.41	0.001
OTHER	0.82	0.66–1.03	0.091
IMI	0.71	0.65–0.78	< 0.001

SDY, *Streptococcus dysgalactiae*; SUB, *Streptococcus uberis*; STA, *Staphylococcus aureus*; STH, human *Staphylococcus aureus;* SAG, *Streptococcus agalactiae*; CNS, coagulase-negative staphylococci; SFA, *Enterococcus faecalis*; CU, contamination; OTHER, minor pathogens including gram-negative bacteria; IMI, intramammary infection; OR, odds ratio; CI, confidence interval.

† , Odds ratio for 2013 versus 2008.

### Within-herd prevalence of *Streptococcus uberis*

**FIGURE 1:**
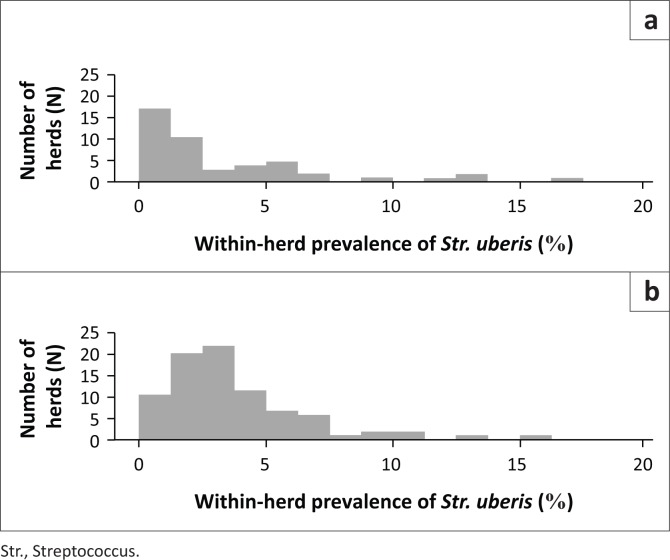
Distribution of the within-herd prevalence (%) of *Streptococcus uberis* in (a) 2008 and (b) 2013.

A total of 43 of 48 herds in 2008 (89.6%; 95.0% CI: 77.3% – 96.5%) and 83 of 87 herds in 2013 (95.4%; 95.0% CI: 88.6% – 98.7%) contained at least one cow that cultured positive for *Str. uberis*. Twelve herds submitted samples in both 2008 and 2013; of these, 10 herds tested positive for *Str. uberis* in both years. For *Str. uberis*-positive herds, the distribution of the within-herd prevalence for each year differed (Figure 1).

The median within-herd prevalence of *Str. uberis* for the combined dairy systems in 2008 and 2013 was 1.72% and 3.10%, respectively (Table 3). The count ratio (CR) for 2013 relative to 2008 was 1.75 (95.0% CI = 1.35% – 2.26%), indicating a 1.75-fold increase in the within-herd prevalence over the period (*p* < 0.001).

**TABLE 3:** Comparison of median within-herd prevalence in *Streptococcus uberis*-positive herds in 2008 (*n* = 48) and 2013 (*n* = 87).

Year	Median %	IQR	CR	95 % CI	*p*
2008	1.72	0.88–5.00	1.75	1.35–2.26	< 0.001
2013	3.10	1.72–4.70			

IQR, interquartile range; CR, count ratio; CI, confidence interval.

**FIGURE 2:**
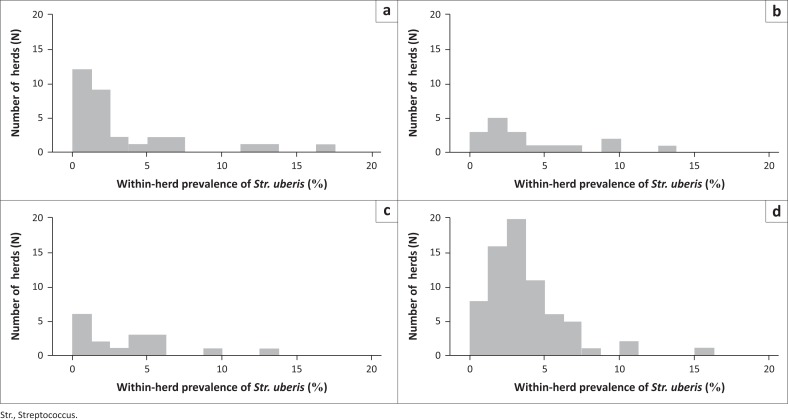
Distribution of the within-herd prevalence of *Streptococcus uberis* (%) for (a) total mixed ration 2008, (b) total mixed ration 2013, (c) pasture 2008 and (d) pasture 2013.

The distribution of the within-herd prevalence for the four different year-system groups differed (Figure 2), with PAS 2013 having the highest concentration of herds (*n ±* 20), with approximately 3% within-herd prevalence for *Str. uberis*.

Despite the lack of statistical evidence for interaction between the system and year (*p* = 0.181), in order to further investigate differences, separate Poisson regression models were used within each year to compare the within-herd prevalence of *Str. uberis* between PAS and TMR dairies (Table 4). The prevalence was slightly higher in the PAS dairies than the TMR dairies during both years; however, the difference was significant only in 2013.

**TABLE 4:** Comparison of the median within-herd prevalence in *Streptococcus uberis-*positive herds in different systems within the same year.

Year	PAS			TMR			CR‡	95 % CI	*p*
	Median %	IQR†	*N*§	Median %	IQR†	*N*§			
2008	2.57	0.99, 5.31	17	1.55	0.69, 4.17	31	1.11	0.89–1.39	0.34
2013	3.35	1.78, 4.59	70	2.59	1.37, 6.02	17	1.17	1.02–1.35	0.02

TMR, total mixed ration; PAS, pasture; IQR, interquartile range; CR, count ratio; CI, confidence interval.

† , Interquartile range;

‡ , Count ratio for PAS versus TMR, estimated using Poisson regression;

§ , Number of herds.

Similarly, separate Poisson regression models were used within each dairy system to compare the within-herd prevalence of *Str. uberis* between years (Table 5). Although there was an increase in prevalence from 2008 to 2013 in both dairy systems, it was only significant in the TMR dairies (*p* < 0.001).

**TABLE 5:** Comparison of the median within-herd prevalence of *Streptococcus uberis*-positive herds in similar dairy systems within different years.

System	2008			2013			CR‡	95 % CI	*p*
	Median %	IQR†	*N*§	Median %	IQR†	*N*§			
TMR	1.55	0.69, 4.17	31	2.59	0.37, 6.02	17	2.13	1.50–3.03	< 0.001
PAS	2.57	0.99, 5.31	17	3.35	1.78, 4.59	70	1.38	0.94–2.01	0.10

IQR, interquartile range; CR, count ratio.

† , Interquartile range;

‡ , Count ratio for PAS versus TMR, estimated using Poisson regression;

§ , Number of herds.

## Discussion

### Prevalence of mastitogenic pathogens

Changing trends in the prevalence of contagious and environmental pathogens, as a cause of mastitis in dairy herds, have been noticed over the last few decades worldwide. Similar subtle changes in pathogen prevalence in South Africa have been noticed (Petzer et al. 2009). The findings in this study are in line with a recent review by Motaung et al. (2017) on mastitis in the African context, which mentions that *S. aureus, S. agalactiae, Str. dysgalactiae, Str. uberis* and *E. coli* are the most commonly reported pathogens in Africa.

In the study by Petzer et al. (2009), quarter and composite cow milk samples were submitted for the identification of pathogens (including partial herd tests) and therefore did not allow estimates of prevalence in the general population. In this study, the prevalence of pathogens was determined from single routine complete herd batches of composite cow milk samples submitted by dairies. Therefore, interpreting a larger timeframe of bacterial trends in South African dairies should be done with caution as partial herd tests, included in the Petzer et al. study (2009), would have investigated specific outbreaks of mastitis and therefore might have overestimated the prevalence of that specific pathogen. Although routine whole herd sampling (lactating group) was performed in this study, the possibility exists that dairy farms that had previous outbreaks of mastitis, as a result of a particular pathogen, subsequently decided to regularly submit routine whole herd milk samples to monitor udder health in the herd. These samples would not have been excluded as a partial herd test as the whole lactating group was tested. Not all dairies were involved in proactive udder health management programmes and therefore did not submit routine samples on a regular basis. These dairies would then have been excluded from the study, subsequently resulting in the selection of dairies that were interested in udder health programmes with improved udder health status and therefore excluding dairies that had mastitis problems at that stage.

The prevalence of *Str. uberis* increased between 2008 and 2013. This is consistent with the findings of Petzer et al. (2009) that highlighted *Str. uberis* emerging as a potentially important pathogen. From this study, it was noticed that the major increase in *Str. uberis* prevalence occurred in PAS herds in 2013. No studies have been conducted in South Africa to investigate the within-herd prevalence of *Str. uberis*. The within-herd prevalence of *Str. uberis* was higher in 2013 compared to 2008, with the median within-herd prevalence of *Str. uberis* higher in 2013 compared to 2008. Although no specific risk factors have been studied, it would be worthwhile investigating the effect that the increased spreading of effluent on pastures has had on the prevalence of *Str. uberis*, because this practice has been favoured in the last few years in PAS dairies. A study by Lopez-Benafides et al. (2007) found that effluent spread on pastures contained high numbers of *Str. uberis*; the numbers declined over time, but, depending on the season of sampling, it could still be detected up to two weeks after spreading. Similar results were observed when samples for *Str. uberis* numbers were taken after cows were removed from grazed pastures (Lopez-Benavides et al. 2007). As discussed by Hogan and Smith (2012), it is important to realise that increased stocking density and stock turnover on pasture will potentially increase the concentration of pathogens.

In a study conducted by Petrovski et al. (2009) in New Zealand, mostly on PAS dairies, *S. aureus* was isolated from 23.7% and *Str. uberis* from 23.3% of all clinical mastitis cases (data July 2005–May 2006, Northland region). Petrovski et al. (2011) did a similar study comparing culture results from all milk samples submitted to five laboratories in New Zealand from August 2003 to December 2006. The findings were consistent with the previous study, with *Str. uberis* (23.6%) and *S. aureus* (23.5%) being the two bacteria most commonly isolated. These findings indicated the importance of *Str. uberis* in PAS dairies in New Zealand; this should be taken note of in South African PAS dairies in future, because of the different control measures that need to be implemented for environmental pathogens (Smith 1983; Smith & Hogan 1993).

Coagulase-negative staphylococci were the most prevalent group of bacteria in this study. This is in agreement with the findings of Petzer et al. (2009). Research conducted in Finland showed similar results, with CNS having the highest prevalence (Pitkälä et al. 2004; Pyörälä & Taponen 2009). In a Norwegian study, CNS (3.3%) and *S. aureus* (8.2%) were the two major bacteria cultured (Østerås, Sølverød & Reksen 2006). A review by Vanderhaeghen et al. (2015) discussed the different CNS species that commonly cause IMI. They indicated that molecular identification methods are very important in understanding the epidemiology of CNS, because *S. epidermidis* and *S. haemolyticus* are common human-adapted pathogens.

As noted by Petzer et al. (2009), *S. aureus* is still one of the major pathogens of concern in South African dairies, because of its chronicity. In this study, *S. aureus* showed a slight decline in prevalence between 2008 and 2013. The specific reasons for this decline could not be identified. Bradley (2002) concluded that a decline in the prevalence of contagious pathogens was seen over a 40-year period in the United Kingdom. A possible reason for this decrease in prevalence of contagious pathogens could be ascribed to improved control methods specifically targeting the contagious pathogens. In contrast to the studies by Petzer et al. (2009), Bradley (2002) and this study, *S. aureus* is still the major mastitis-causing pathogen found in other countries. In a Norwegian study, *S. aureus* was the most prevalent pathogen cultured from 8.2% of all samples (Østerås et al. 2006).

*Streptococcus agalactiae* showed a marked increase in prevalence in TMR dairies in 2013. This is in agreement with Petzer et al. (2009) who also reported marked increases in prevalence in 2001 and 2003. These peaks were ascribed to large outbreaks of *Str. agalactiae* mastitis cases. Although this is difficult to prove from the current data, it is also likely that the peak seen in 2013 in TMR dairies could be because of several outbreaks of *Str. agalactiae* in South African dairies (I.M. Petzer [University of Pretoria] pers. comm., 2015). *Streptococcus agalactiae* is a highly contagious obligate intramammary pathogen and is one of the major causes of subclinical mastitis with a potentially high cure rate after antimicrobial therapy and well - managed sanitary procedures (Keefe 2012; Smith & Ward 1975). As a result, farms become free from *Str. agalactiae* (Andersen et al. 2003; Barkema et al. 2009) with a subsequent decrease in the prevalence of *Str. agalactiae*. However, if on-farm biosecurity is lacking and newly purchased infected cows are introduced to the farm, the rapid spread of IMI because of *Str. agalactiae* is possible (Barkema et al. 2009).

### Limitations of the study

This study was conducted using samples submitted to the OML and was therefore not based on random sampling from the entire South African dairy population. This could have resulted in several sources of selection bias which may have influenced the prevalence estimates. Herd sizes were not always known; therefore, it was not in all instances possible to determine whether a farm submitted routine whole herd samples. Herd size was determined through historical data, and if this was deemed to be insufficient, the herd was excluded, but it is unlikely to have biased the results.

Although the methods for culturing and identifying the bacteria in the laboratory stayed relatively constant from 2008 to 2013, other procedures such as collection methods, personnel collecting the samples and travel distances (time) differed during the two years. This may have resulted in slightly different sensitivities for detecting pathogens between the two years, although this is unlikely to have significantly biased the results.

The small sample sizes in some comparison groups likely resulted in reduced statistical power to detect differences. This might have been the case when the within-herd prevalence of *Str. uberis* was compared between TMR- and PAS-based dairies during 2008 and 2013. It was noted that there were only 17 PAS-based dairies in 2008 and 17 TMR-based dairies in 2013 used in the study. This may have resulted in decreased power of the statistical testing in the study, taking into consideration that there are over 1300 registered dairy farmers in South Africa. In the case of only 17 PAS- and TMR-based dairies, it may be that farms with extremely high or low within-herd prevalence of *Str. uberis* were over-represented, resulting in biased results.

The total populations of TMR- and PAS-based dairies in 2008 and 2013 are not known; therefore, it is not known whether they were proportionally represented in the samples used; if not, this could have resulted in bias in the estimate of the overall prevalence. Furthermore, a possible reason for the overall increasing numbers of farms during 2013 is because of the increasing demand of dairy producers to enrol in the proactive udder health approach promoted to producers.

## Conclusion

Differences in the prevalence of the mastitogenic pathogens were shown between the different years and dairy systems.

Coagulase-negative staphylococci were the most prevalent pathogens in both TMR and PAS dairies during 2008 and 2013. *Staphylococcus aureus* showed an overall decrease in prevalence between 2008 and 2013. *Streptococcus agalactiae,* however, showed a steep increase in prevalence during the same period, potentially because of localised outbreaks. *Streptococcus uberis* showed a significant increase in prevalence in this study, both overall and within affected herds, with the highest prevalence in PAS dairies during 2013.
